# Wanted! Health information students & early career health information workers new to writing for publication

**DOI:** 10.1111/hir.12455

**Published:** 2022-08-03

**Authors:** Frances Johnson

**Affiliations:** ^1^ Department of Languages, Information & Communications Manchester Metropolitan University Manchester UK

**Keywords:** dissemination, dissertations, evaluation, information services, research and development

## Abstract

Invitation to health information students and early career health information workers new to writing for publication to share evaluations of existing services or investigations into service improvement.

The purpose of the *Dissertations into Practice* Regular Feature to two‐fold. First, to encourage health information students and new and early career health information workers to write for publication. Second, to highlight the potential impact of student and early career health information worker's projects for practice.

The scope of this Regular Feature is broad in that, provided it is related to the provision of health information or health library services, any topic or approach can be considered for inclusion. With an emphasis on sharing practice, contributions can be from any sector relevant to the delivery of health information including public libraries. Recent submissions have fallen into two broad categories: evaluations of existing services or investigations into service improvement.

Best of all, is that contributions can be short (a maximum of 2500 words!), are not peer reviewed, and authors are fully supported in bringing their writing to publication. Interested? For further information please contact Frances Johnson, *Dissertations into Practice* Regular Feature Editor at f.johnson@mmu.ac.uk or send a direct message to @HILJnl at https://twitter.com/HILJnl.

